# Parametrically optimized feather degradation by *Bacillus velezensis* NCIM 5802 and delineation of keratin hydrolysis by multi-scale analysis for poultry waste management

**DOI:** 10.1038/s41598-022-21351-9

**Published:** 2022-10-12

**Authors:** Isha Sharma, Kumar Pranaw, Hemant Soni, Hemant Kumar Rawat, Naveen Kango

**Affiliations:** 1 Department of Microbiology, Dr. Harisingh Gour Vishwavidyalaya (A Central University), Sagar, 470003 Madhya Pradesh India; 2 Department of Environmental Microbiology and Biotechnology, Institute of Microbiology, Faculty of Biology, University of Warsaw, Miecznikowa 1, 02-927 Warsaw, Poland; 3 Section of Microbiology, Central Ayurveda Research Institute (CARI), Jhansi, 284003 Uttar Pradesh India

**Keywords:** Microbiology, Environmental sciences

## Abstract

Enormous amounts of keratinaceous waste make a significant and unexploited protein reserve that can be utilized through bioconversion into high-value products using microbial keratinases. This study was intended to assess the keratinase production from a newly isolated *B. velezensis* NCIM 5802 that can proficiently hydrolyze chicken feathers. Incubation parameters used to produce keratinase enzyme were optimized through the Response Surface Methodology (RSM) with chicken feathers as substrate. Optimization elevated the keratinase production and feather degradation by 4.92-folds (109.7 U/mL) and 2.5 folds (95.8%), respectively. Time-course profile revealed a direct correlation among bacterial growth, feather degradation, keratinase production and amino acid generation. Biochemical properties of the keratinase were evaluated, where it showed optimal activity at 60 °C and pH 10.0. The keratinase was inhibited by EDTA and PMSF, indicating it to be a serine–metalloprotease. Zymography revealed the presence of four distinct keratinases (Mr ~ 100, 62.5, 36.5 and 25 kDa) indicating its multiple forms. NMR and mass spectroscopic studies confirmed the presence of 18 free amino acids in the feather hydrolysates. Changes in feather keratin brought about by the keratinase action were studied by X-ray diffraction (XRD) and spectroscopic (FTIR, Raman) analyses, which showed a decrease in the total crystallinity index (TCI) (1.00–0.63) and confirmed the degradation of its crystalline domain. Scanning electron microscopy (SEM) revealed the sequential structural changes occurring in the feather keratin during degradation. Present study explored the use of keratinolytic potential of the newly isolated *B. velezensis* NCIM 5802 in chicken feather degradation and also, unraveled the underlying keratin hydrolysis mechanism through various analyses.

## Introduction

Keratin waste like feathers, hairs, nails, hooves, horns, wool, etc. can serve as an excellent source of protein. Keratins are insoluble, recalcitrant, highly cross-linked, fibrous structural proteins resistant to proteolysis^1^. The ordered structural arrangement of α-helical and β-sheet configurations provides the stability and robustness, whereas disulfide bonds are equally responsible for conferring a high degree of rigidity, strength, and resistance to chemical as well as biological degradation to the protein. In the poultry processing industry, the increased production of meat over the years has caused concomitant increase in unwanted solid poultry waste, mainly keratin^2–4^. Literature suggests that more than 85% of keratin content is produced in the form of feathers and roughly around 10 million tons per year of feather waste is generated worldwide, representing a massive sustainable protein reserve^5^. However, the high degree of disulfide bonds and hydrophobic nature of keratin makes its utilization and management extremely difficult and bears hazardous consequences upon the environment^6^.

This recalcitrant reserve presents a formidable challenge for its processing into value-added by-products^7^. Conventionally, the keratin biomass is hydrolyzed further by physical and chemical processes such as hydrothermal extraction, or treatment with reducing agents leading to production of stimulants, animal feedstock, fertilizers, amino acids, and soil conditioner^8,9^. Feather meals are commonly obtained by subjecting the keratin biomass to the hydrothermal process where the feathers are cooked at high pressure and temperature. Hydrothermal treatment provides efficient and complete conversion of feathers into feather meal; however, the resulting feather meal loses its nutritional value and digestibility during the process. As the decomposition of feather biomass through a hydrothermal or chemical process, ensues the decomposition of essential amino acids, such as methionine, lysine, tryptophan, cysteine, and tyrosine. Apart from being detrimental to the environment, physico-chemical treatments also need high energy and reagent inputs incurring high costs^8,10^.

Biodegradation of the keratinous waste through microbial keratinases is an effective and inexpensive valorization approach for the efficient treatment of keratin-rich wastes that entails waste management, human and environmental safety, and resource generation (amino acids, peptides, and nonprotein nitrogenous compounds)^11,12^. Also, the conversion of low-priced readily accessible chicken feathers into dietary protein for animal feedstock strengthens a bio-economical approach. Keratinases (EC 3.4./24/99.11) are a set of proteases with a unique keratinolytic ability to depolymerize fibrous, recalcitrant structural keratins into soluble proteins^13^. A number of bacteria, fungi, and actinomycetes are known to produce keratinases^14,15^. Ker A was the first purified keratinase characterized from *Bacillus licheniformis* strain which belonged to subtilisin-like serine protease family (S8)^16^. Some other bacterial keratinases of the S8 family include those from *B. subtilis*, *B. halodurans*, and *Streptomyces* sp.^17^. Furthermore, keratinases belonging to the metalloprotease families have also been identified from various groups of bacteria and fungi^18,19^.

Keratinases have many prospective applications that are linked to the efficient conversion and production of feather meal, animal feedstock, biofertilizers, cosmetics, thermo-bioplastics, personal care items including anti-dandruff shampoo, and pharmaceutical products such as prion decontaminants^20–22^. The sturdiness of bacterial keratinases and the differences in their catalytic proficiency have highlighted the necessity for investigating other hyperactive and versatile keratinase-producing strains.

Therefore, the present study explores a cost-effective and eco-friendly approach to keratin waste recycling and optimization protocol for the enzymatic degradation of feather wastes along with its conversion into value-added products such as amino acids, soluble proteins, and oligopeptides using *B. velezensis* NCIM 5802 keratinase. Multi-scale characterization of the biocatalyst and the substrate was carried out to delineate the mechanism of the efficient keratin degradation.

## Materials and methods

### Chemicals and materials

All the chemicals used in this study were of analytical grade and utmost available purity. Keratin azure was purchased from Sigma-Aldrich, India while casein and gelatin were purchased from HiMedia, India. Chicken feathers were obtained from a local chicken slaughterhouse, extensively washed with warm water and mild detergents to remove blood, dirt, and other impurities followed by sun-drying for 4–5 days; and then were stored at room temperature, for further use.

### Sample collection and isolation

Soil samples were collected from the university campus situated on the Patharia hills, Sagar, MP, India. Using the principle of enrichment culture, the collected samples were inoculated in distilled water containing 1% (w/v) autoclaved, clean intact feathers, and incubated at 37 °C, 120 rpm for two weeks. In cases where complete solubilization of feathers was observed, the suspension was serially diluted and inoculated on feather meal minimal salt agar (FMMSA)^23^. Plates were then incubated at 37 °C for 48 h and single colonies were transferred on FMMSA plates to get pure culture. Pure bacterial cultures, thus isolated, were assayed for extracellular caseinase and gelatinase production on casein or gelatin agar plates^24^. Promising bacterial cultures representing a prominent zone of hydrolysis on casein agar plate were tested for their feather degrading capability using the feather as a sole source of carbon and nitrogen, under submerged conditions at 37 °C and 200 rpm for 48 h. Among these, isolates showing higher feather degradation were further evaluated for feather solubilization (% w/w), soluble protein content, amino acid release, and extracellular keratinase activity. Promising bacterial isolates were sub-cultured and maintained on keratin agar slants (40% w/v) and were stored at 4 °C/− 80 °C for further use.

### Morphological and molecular identification of the strain

Molecular validation of the isolate identity was achieved by 16S rDNA sequence assessment which involved extraction of genomic DNA and amplification and sequencing of 16S rRNA gene. Extraction of genomic DNA was carried out from 18 to 20 h old bacterial culture using Genomic DNA extraction kit (Thermo Fisher Scientific). Amplification of the 16S rDNA was carried out using Applied Biosystems Veriti Thermal Cycler, using two 16S rDNA universal primers with the subsequent sequences: 5′-AGAGTTTGATCCTGGCTCAG-3′ (27F) and 5′-GGTTACCTTGTTACGACTT-3′ (1492R) as forward and reverse primers, respectively. Amplification of the 16S genes was carried out as follows: initial denaturation 98 °C for 5 min, denaturation 95 °C for 30 s, annealing 55 °C for 30 s, extension 72 °C for 90 s, and final extension 72 °C for 8 min and the PCR was run for 30 cycles^5,25^. The amplified PCR products were sequenced, the resulting sequence was compared with the available sequences in the GenBank using BLAST tool and deposited in the GenBank. The culture was deposited in National Centre for Industrial Microorganism (NCIM), Pune. The isolate was identified by phylogenetic clustering and tree was constructed using ClustalW. Morphological features of NCIM 5802 were also studied by scanning electron microscopy (SEM).

### Inoculum preparation

Inoculum broth (50 mL) containing (g/L): 10.0 glucose, 10.0 peptone, 3.0 yeast extract, 2.0 calcium chloride (pH 7.0) was inoculated with a loopful of bacterial suspension and was incubated overnight at 37 °C, 150 rpm.

### Keratinase production by NCIM 5802

Keratinase production was carried out in the feather meal minimal salt broth (FMMSA devoid of agar) supplemented with 0.5% (w/v) chicken feathers at 200 rpm at 40 °C for 96 h. After fermentation, the remaining chicken feathers were removed from the cultivation fluid by filtration through a glass filter. The culture filtrate thus obtained was centrifuged at 10,000*g* for 10 min at 4 °C. The protein was precipitated using acetone as described earlier^23^. The protein precipitate was resuspended in 0.05 M Citrate buffer (pH 5.0), dialyzed using 10 kDa cut-off membrane and was used as the source of keratinase.

### Keratinase assay

Keratinase activity was performed according to the method described by Navone and Speight^26^ using keratin azure as the substrate with some modifications. Concisely, 0.01 g of keratin azure was suspended in 0.05 M Tris–HCl buffer (pH 10.0) to which appropriately diluted enzyme was added. Contents were mixed thoroughly by vortexing and the tubes were incubated at 40 °C for 1 h in water bath. After incubation, the tubes were centrifuged at 10,000*g* for 5 min and the absorbance of the clear supernatant was taken at 595 nm against the appropriate blank. One unit (U) of keratinolytic activity was defined as the increase in absorbance by 0.01 at 595 nm under the standard assay conditions^27,28^**.**

### Optimization of keratinase production and feather degradation using response surface methodology (RSM)

The optimized process parameters for keratinase production (U/mL) and feather degradation (%) by OVAT were taken and four independent variables (A) Temperature (℃), (B) pH, (C) Substrate concentration (%) and (D) Inoculum size (%) were further optimized by Central Composite Design (CCD) using Design-Expert v13-Stat-Ease software. A plot of total 30 experiments based on the randomized factorial design consisted of five levels (− α, − 1, 0, + 1, + α) with four variables, and 6 replicate trials of central points. These experiments were performed to see the response pattern and to ascertain the optimum combination of factors. The response of the design was measured in terms of keratinase activity (U/mL) and feather degradation (%). The experimental data were fitted to a second-order polynomial equation through multiple regression analysis, and analysis of variance (ANOVA) indicated the competence of the model so obtained. Mutual interactions between the independent variables were characterized by three-dimensional response surface plots^29^.

### Time course profile of keratin degradation

Time-dependent study of feather keratin degradtion by *B. velezensis* NCIM 5802 was carried out for 96 h in 500 mL Erlenmeyer flasks under the optimized process conditions (3.5% inoculum, 0.625% substrate concentration at 40 °C and 200 rpm). Aliquots were withdrawn from the flasks at regular time intervals (12 h) and were tested for various parameters, such as, bacterial growth, keratinase activity (U/mL), residual substrate (%), protein concentration (µg/mL), amino acid content (µg/mL), pH, and soluble oligopeptides (µg/mL).

#### Growth pattern of NCIM 5802

During fermentation, the growth pattern of the bacterium was studied by measuring the increase in absorbance at 600 nm up to 96 h. All the experiments were performed in triplicates and the data represents average ± SD.

#### Protein content

The soluble protein content in the fermentation medium, was measured by Bradford method with bovine serum albumin (BSA) as standard ^30^.

#### Amino acid content

Total amino acid content present in the feather hydrolysate was estimated using the ninhydrin method. In a nutshell, 30 μL of the hydrolysate and 150 μL of phosphate citrate buffer (pH 5.0) were mixed thoroughly, to which 3% ninhydrin solution was added (1:1) and the mixture was vortexed for 1 min and subjected to boiling for 15 min. Upon the development of color, the reaction was stopped by cooling the tubes on ice. Following this, solution of isopropanol and water (7:3) was added to the mixture and after vortexing the absorbance was measured at 570 nm with serine as standard^31^.

#### Soluble oligopeptides in the hydrolysate

To study the soluble oligopeptide content in the aliquots, the samples were centrifuged at 5000*g* for 10 min and the hydrolysates were passed through 10 kDa cut-off Amicon Ultra membrane (Merck Millipore, India). Later, the absorbance of the filtrate was measured at 230 nm^29^.

#### Feather weight loss

Biodegradation of substrate by NCIM 5802 during the course of fermentation was evaluated in terms of substrate weight loss (%). After fermentation, the feather residues were separated and collected from the culture supernatant by filtration. The collected residual feathers were then washed thoroughly and dried in a hot air oven at 100 °C for 48 h. Biodegradation of feathers was expressed as percentage weight loss with respect to the initial dry weight of the substrate (before and after incubation). The uninoculated flask with feathers was used as control for the assessment.

1 $${\text{Feather degradation }}\left( {\text{\% }} \right) = \frac{{{\text{ Initial weight}} - {\text{Final weight}}}}{{\text{Initial weight}}}{ } \times { }100$$

### Enzyme characterization

The keratinase enzyme produced by NCIM 5802 was analyzed by studying the effect of temperature and pH on the enzyme activity and stability. In addition, the effect of some metal ions, surfactants, inhibitors, and activators was also investigated.

#### Effect of pH and temperature on keratinase

To study the effect of pH, keratinase activity was performed by incubating the reaction mixture at different pH (4.0–11.0) for 1 h. Keratin azure (1% w/v) was dissolved in different buffers (0.05M) viz. citrate buffer (pH 4.0–5.5), phosphate buffer (pH 6.0–8.5), glycine–NaOH buffer (pH 9.0–10.0), and NaOH-potassium dihydrogen phosphate buffer (pH 11.0). The enzyme activity at different pH values was expressed in terms of relative activity (%). The effect of temperature on keratinase activity was studied by incubating the reaction mixture at different temperatures ranging from 30 to 90 °C in 0.05 M glycine–NaOH buffer (pH 10.0) with 1% (w/v) keratin azure as substrate^32^. The residual keratinase activity was expressed as the relative enzyme activity with respect to control.

#### Effect of pH and temperature on keratinase stability

To examine the pH stability, keratinase preparation was pre-incubated with an equal amount of different buffers viz. phosphate buffer (pH 6.0–8.5), glycine–NaOH buffer (pH 9.0–10.0), and NaOH-potassium dihydrogen buffer (pH 11.0), respectively, for 80 min at 60 °C. Aliquots were withdrawn at regular time intervals and the residual keratinase activity was estimated using keratin azure as substrate as described above. The thermostability of the enzyme was assessed by incubating the keratinase at different temperatures (60–90 °C) for 80 min at pH 10.0 (Glycine–NaOH buffer). Aliquots were withdrawn at regular intervals and the residual keratinase activity was determined. The relative enzyme activity was defined as the percentage of the ratio between the keratinase activity of the treated sample and the activity present in the untreated control against a suitable blank.

#### Effect of metal ions, detergents, solvents, activators, and inhibitors

To study the effect of metal ions on NCIM 5802 keratinase, the keratinase activity was investigated in the presence of monovalent (Na^+^ and K^+^) and divalent metal ions (Cu^2+^, Ca^2+^, Mg^2+^, Hg^2+^, Zn^2+^, Fe^2+^, Ag^+^, NH_4_^+^, Mn^2+^). The effect of some activators and inhibitors such as urea, EDTA (ethylenediaminetetraacetic acid), β-ME (β-mercaptoethanol) and PMSF (phenylmethanesulfonyl fluoride) was also tested. The influence of certain surfactants and solvents on keratinase was tested using Triton X-100, Tween 80, SDS (sodium dodecyl sulfate), ethanol, methanol, and glycerol (0.1, 0.5, 1, 2 and 5%). The enzyme was preincubated with metal ion, detergent, solvent, activator or inhibitor for 30 min at 28 °C and the residual relative activity (%) was calculated against the appropriate control.

### Multi-scale sequential analysis of feather degradation

#### Surface topology of degraded feather

To study the structural changes occurring during feather hydrolysis, feather samples (collected at 12 h interval up to 96 h) from the culture were visualized using scanning electron microscope (SEM) as described earlier^23^. Samples were air-dried and sliced into small fragments before fixing on the sample holder. The sample holder stubs were exposed for 60 s to the Denton vacuum sputter coater for gold coating and were examined with FEI Nova NanoSEM 450 (FEI, USA) at 15 kV.

#### Fourier-transform infrared (FTIR) spectroscopic analysis

The presence of characteristic functional groups in the untreated and hydrolyzed feathers was ascertained by FTIR analysis^33^. The changes occurring in the functional groups of the keratin were observed in the feather samples. The FTIR spectra was obtained using an ALPHA II spectrometer, (Bruker) in the spectrum range 4000–500 cm^−1^ with 40 scans at a resolution of 4 cm^−1^.

#### Raman spectroscopic studies of keratinase treated feathers

To study keratinase mediated feather solubilization process, Raman spectra of the residual feather mass obtained after the fermentation process was studied. Samples of hydrolyzed feathers were taken at regular time intervals and were air-dried. The inVia confocal Raman spectroscope (Renishaw) was used to record the spectra of the samples placed on a glass slide and focused using a video camera through the Raman option at 785 nm with a maximum output of 250 mW and 100 µm size of the laser spot. To get the optimal Raman spectra, the scan was repeated 4 times in the spectrum ranging from 3000 to 500 cm^−1^ with 40 scans^34^. For comparison, the spectrum of the feathers collected from the uninoculated flask were used as control.

#### X-ray diffraction (XRD) analysis

The X-ray diffraction (XRD) pattern of both degraded chicken feathers and intact feathers was conducted using X-ray diffractometer (Bruker D8 ADVANCE, Germany). The crystallinity was observed by adjusting the monochromator diffraction beam coupled with a copper X-ray tube at 40 kV and 30 mA, while the diffraction spectra were signified by the 2*θ* angle^35^. The scattering angle (2*θ*) was from 6 to 26° at a scanning rate of 2° min^−1^. The crystallinity index (CrI) was calculated using the intensities of amorphous (*I*_*am*_) as well as crystalline regions as given below:

2 $$CrI = \frac{{I_{002} - I_{am} }}{{I_{002} }} \times 100$$

where, the highest diffraction intensity at the highest peak was *I*_002_ (~ 21.2) and the lowest diffraction intensity between the major and secondary peaks was *I*_*am*_ (~ 14.0).

The average crystallite size of the native and degraded feather was determined using the Scherrer’s equation:

3 $$D_{{{\text{SE}}}} = \frac{{ {\text{k}}\lambda }}{\beta \cos \theta } \times 100$$

where *D*_SE_ = crystallite size (Å), κ = Scherrer’s constant (0.9), λ = 1.5406 Å (wavelength of X-ray radiation), β = full width at half maximum (FWHM) of X-ray diffraction peaks and θ = Bragg’s angle (angle of diffraction) relating to the planes.

#### Amino acid profile of feather hydrolysates

The amino acids in the feather hydrolysate samples (collected periodically after 12 h of incubation over 96 h) were analyzed through Electrospray Ionization-Mass Spectrometry (ESI–MS). Mass spectra of the samples were recorded in using Xevo G2-S QTOF mass spectrometer in positive ion mode paired with a 2424 Evaporative Light Scattering (ELS) detector. Collision induced dissociation (CID) was caused by Helium and the generated data was assessed using MassLynx data analysis software (Version 4.1). Uninoculated sample was taken as control and standard amino acids were taken as a reference to assign the peaks of the experimental samples^30^.

#### NMR spectroscopic studies

NMR analysis of the feather hydrolysate was carried out to reveal the sequential changes brought about by the keratinase action on feathers. For this, samples were prepared in HPLC grade water and small amounts of deuterium oxide (D_2_O) and tetramethylsilane (TMS) were added for spin locking and internal reference, respectively. The ^1^H was decoupled with the SPINAL-64 ^1^H decoupling sequence. ^1^H liquid state NMR experiment of feather hydrolysates was carried out using a liquid state NMR probe. The water suppression was done by pre-saturation method and the uninoculated medium was taken as a control for all the ^1^H NMR experiments in the liquid state^36^.

#### Keratinase zymography

Semi-native SDS-PAGE was carried out using 12% (w/v) polyacrylamide gel as described earlier^33^ with slight modification. For keratinase zymography, 20 µL of appropriately diluted enzyme sample was loaded on the semi-denaturating gel containing soluble keratin (0.1% w/v). Electrophoresis of the gel was conducted for ~ 3 h at 90 V at 4 °C. Afterwards, the gel was removed and incubated at 40 °C for 24 h and the presence of keratinase was visualized by staining with Coomassie brilliant blue G-250. The active bands on the gel appeared as the zone of keratin hydrolysis.

### Quality assurance and quality control

All the experimental methods and procedures mentioned were conducted as per the standard operating protocols and good laboratory practices. All the chemicals used were of analytical grade with the highest available purity and the machines used for analysis were calibrated. The experimental setup was carried out in triplicates and the results were expressed as mean ± SD.

## Results

### Morphological and molecular identification of the isolate

Based on the distinct zone of hydrolysis on casein and keratin agar plates, a potent keratinolytic bacterium NCIM 5802 was selected for this study. The isolate degraded keratin (40.2%) and produced high titers of extracellular keratinase (22.3 U/mL) accompanied with the release of amino acids (203.05 μg/mL) and soluble protein content (62.2 μg/mL). The 16S rDNA (ON203026) of NCIM 5802 revealed that this isolate belonged to the genus *Bacillus* having the highest homology with *B. velezensis* (97.38% homology; accession number: OM074020) and *B. tequilensis* (97.34% homology; accession number: OM061696.1) (Supplementary Fig. S1). Staining followed by the microscopic observation confirmed it to be a gram-positive bacillus. The DNA G + C content (https://en.vectorbuilder.com/tool/gc-content-calculator.html) of the strain NCIM 5802 was found to be 55.14 mol%. The strain was deposited in the National Collection of Industrial Microorganisms (NCIM), Pune, India and has been assigned accession number NCIM 5802.

### Production optimization of the process parameters for waste feather degradation

To attain the optimal response for maximizing keratinase production for feather degradation, four significant independent variables [X1: Temperature (℃), X2: pH, X3: Substrate conc. (%) and X4: Inoculum size (%)] were selected and two responses, R1: Keratinase activity (U/mL) and R2: Feather degradation (%) were generated. All the experiments were conducted in triplicate, and the obtained results represent average ± SD.

#### Response surface optimization of feather degradation and keratinase production

**Figure 1:**
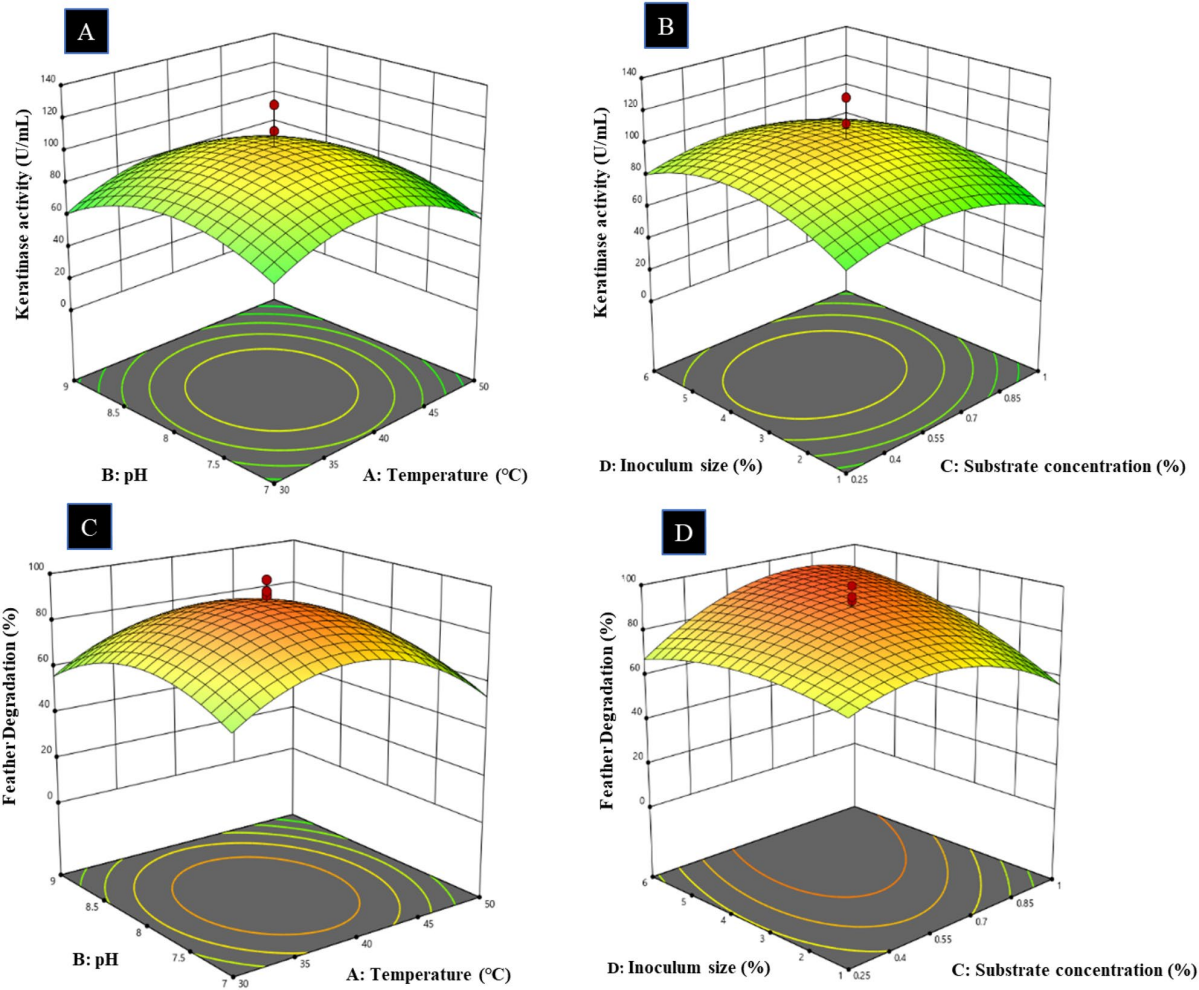
Response surface 3D plots showing the interaction of factors affecting **keratinase production** where, (**A**) pH and temperature (°C), (**B**) inoculum size (% v/v) and substrate concentration (%) and **feather degradation** where, (**C**) pH and temperature (°C), (**D**) inoculum size (%) and substrate concentration (%).

The four variables were analyzed using the central composite design (CCD) approach, and the corresponding percentage of substrate degradation and enzyme production (U/mL), along with the interactional relationship among the selected variables was observed in terms of coded values. The optimal values of the four independent variables obtained from the maximum polynomial model point were found to be 40 °C, 8.0, 0.625%, and 3.5% for temperature, pH, substrate concentration and inoculum size, respectively, with the predicted keratinase production and feather degradation values being 102.9 U/mL and 90.7%, respectively, while actual experimental values were different. In addition, 104.5, 100.9, 128.3, 110.5, 101.7 and 112.5 keratinase activity (U/mL) and 93.4, 100, 97.2, 94.3, 94.6 and 95.2% feather degradation data was observed in the run numbers 12, 13, 20, 23, 28 and 30, respectively (Supplementary Table S1). The obtained result at p = 0.0005 indicated that there is a statistically significant relationship between the studied variables and the measured response, namely enzyme production (U/mL) and substrate degradation (%). Additionally, the obtained value of the determination coefficient (R^2^ = 0.964), which is a measure of the fitting degree for the applied model, indicated that about 3.6% of the total variations in the measured response are not explained by the model. Figure 1 presents the interactive effects of independent variables for acquiring the maximum and optimal level of the studied responses, namely keratinase yield and substrate degradation as presented by three-dimensional (3D) surface and contour plots. The F-value of the model was 7.07 and 27.16 for keratinase production and residual keratin substrate, respectively with p-values less than 0.0500, indicating that the present model is significant and noteworthy. In this context, the current study revealed the high efficiency of NCIM 5802 keratinase in feather keratin degradation. Parametric optimization of feather degradation and keratinase production was successfully applied leading to 2.5 and 4.92-fold enhancement, respectively.

### Time course profile of keratin degradation

**Figure 2:**
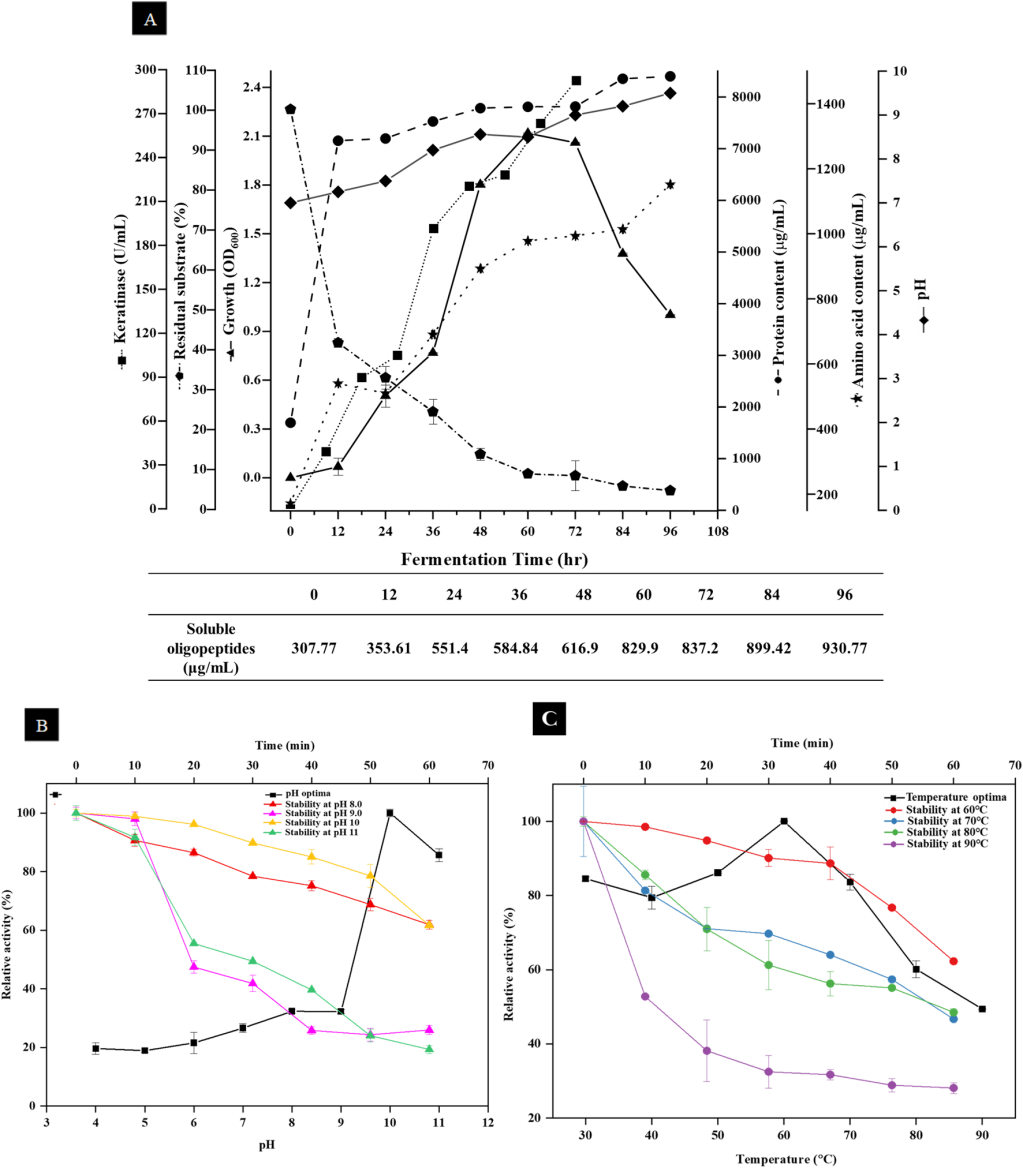
Time course profile of keratinase production (**A**) time course of decomposition of chicken feathers by *B. velezensis* NCIM 5802 (%), keratinase activity (U/mL), bacterial growth (OD_600_), soluble protein content (μg/mL), amino acid content (μg/mL) and pH (optimized culture conditions: initial pH 7.0, time 96 h, 3.5% v/v inoculum, 0.625% w/v substrate concentration at 40 °C and 200 rpm). Temperature and pH profile of keratinase produced by *B. velezensis* NCIM 5802 (**B**) pH optima and stability (**C**) temperature optima and stability. Values represent average of three independent replicates ± standard deviation.

Time course profile was studied for 96 h of cultivation to understand the relationship among growth curve of NCIM 5802 and keratinase production (U/mL), pH alteration, residual substrate concentration (%), protein concentration (μg/mL), amino acid (μg/mL), and oligopeptide production (μg/mL) under RSM optimized conditions (Fig. 2A). Keratinase production by NCIM 5802 reached highest (292.6 U/mL) after 96 h of fermentation in the late stationary phase of its growth. The residual feather was found to be 1.81% (w/v) accompanied with the increase in soluble protein (8398.5 μg/mL), oligopeptides (930.77 μg/mL), amino acids (1151.8 μg/mL) and a pH shift from 7.0 to 9.5.

### Characterization of the NCIM 5802 keratinase

#### Effect of pH and temperature

The activity profile of the keratinase indicated that it exhibits a broad pH range with an optimal activity at 10.0 (Fig. 2B). The data presented in Fig. 2C shows the optimal temperature of the keratinase to be 60 °C; hence, the enzyme can be categorized as an alkali and thermo-stable keratinase. The results presented in Fig. 2B suggested that the enzyme was stable at pH 10. It retained more than 60% activity up till 60 min indicating its tolerance for alkaline pH. However, it lost about half of its activity at pH 11.0 in 30 min (49.43%). The data revealed that the keratinase had optimal activity at 60 °C and remained stable at 60 °C for up to 40 min (90.13%), while the half-life of keratinase at 80 °C was 60 min (Fig. 2C).

#### Effect of metal ions, detergents, solvents, surfactants, activators, and inhibitors

Among various metal ions, keratinase activity was positively influenced in the presence of Ca^2+^ (153.8%) and Mg^2+^ (211.8%) (Table 1). Metal ions such as Cu^2+^, K^+^, Na^+^, Hg^2+^, Zn^2+^, Fe^2+^, Ag^+^, NH_4_^+^ and Mn^2+^ were found to be inhibitory.

**Table 1:** Effect of various metal ions, inhibitors, reducing agents, and solvents on the activity of *B. velezensis* NCIM 5802 keratinase.

Tested	Metal ions at different concentration				
	1 mM	5 mM	10 mM	15 mM	20 mM
**Residual activity of keratinase % (mean ± SD)**					
Control	100 ± 0.1	100 ± 0.1	100 ± 0.05	100 ± 0.1	100 ± 0.1
Cu^2+^	99.1 ± 0.1	78.2 ± 0.6	56.5 ± 0.3	52.4 ± 1.5	37.6 ± 0.3
K^+^	77.6 ± 0.8	58.2 ± 5.5	57.0 ± 0.6	57 ± 4.2	55.9 ± 0.3
Na^+^	84.4 ± 0.7	76.9 ± 1.1	68 ± 2.4	49 ± 3.7	42.9 ± 3.22
Ca^2+^	153.8 ± 0.18	184.5 ± 0.8	138.5 ± 4.5	138.7 ± 0.5	88.8 ± 0.8
Mg^2+^	211.8 ± 1.3	191.2 ± 1.4	159 ± 1.13	70.8 ± 1.7	67.0 ± 0.2
Hg^2+^	59.3 ± 0.8	51.9 ± 0.7	42.3 ± 1.6	41.7 ± 0.16	38.9 ± 0.15
Zn^2+^	52.3 ± 0.3	49.6 ± 0.3	35.9 ± 1.6	36.1 ± 5.1	27.0 ± 2.7
Fe^2+^	58.7 ± 0.7	40 ± 0.4	125 ± 0.9	18.8 ± 0.25	13.9 ± 0.07
Ag^+^	66.7 ± 0.27	65.5 ± 0.12	62.0 ± 0.12	56.3 ± 0.62	55.8 ± 0.43
NH_4_^+^	68.8 ± 0.8	60.2 ± 0.4	52.8 ± 0.6	37.9 ± 0.2	15.4 ± 0.6
Mn^2+^	32.7 ± 0.70	40.1 ± 0.5	53.8 ± 2.4	43.4 ± 0.4	36.02 ± 1.01
NaN_3_	66.4 ± 0.94	57.2 ± 0.2	53.3 ± 0.7	22.5 ± 1.5	18.2 ± 0.03

Tested	Activators/ inhibitors at concentration				
	1 mM	5 mM	10 mM	15 mM	20 mM
**Residual activity of keratinase % (mean ± SD)**					
Control	100 ± 0.1	100 ± 0.12	100 ± 0.5	100 ± 0.1	100 ± 0.1
Urea	30.9 ± 1.07	38.3 ± 0.4	19.1 ± 0.12	10.5 ± 0.45	5.5 ± 1.31
EDTA	52.7 ± 2.8	51.4 ± 0.52	40 ± 0.12	31.9 ± 2.2	32.1 ± 1.2
B-ME	46.4 ± 1.2	41.9 ± 0.5	36 ± 0.5	35.5 ± 0.87	28.9 ± 2.1
PMSF	7.8 ± 1.18	8.6 ± 0.5	6.2 ± 0.13	6.2 ± 0.7	5.3 ± 0.15

Tested	Detergents, surfactants, and solvents at concentration				
	0.1%	0.5%	1%	2%	5%
**Residual activity of keratinase % (mean ± SD)**					
Control	100 ± 0.1	100 ± 0.12	100 ± 0.05	100 ± 0.1	100 ± 0.1
Tween-80	26.7 ± 0.43	28.1 ± 0.6	5.6 ± 2.12	5.0 ± 0.2	2.5 ± 1.5
Triton- X 100	35.3 ± 1.14	71.1 ± 0.6	72.6 ± 2.3	37.0 ± 0.3	33.9 ± 0.3
SDS	38.2 ± 0.43	43.9 ± 0.4	41.3 ± 0.11	27.5 ± 0.8	17.5 ± 4.6
Methanol	26.9 ± 1.14	22.1 ± 0.3	21 ± 0.12	13.8 ± 0.7	11.4 ± 0.8
Ethanol	27.1 ± 0.4	34.1 ± 3.30	23.6 ± 0.25	18.2 ± 3.1	15.5 ± 0.05
Glycerol	29.1 ± 1.1	34.8 ± 0.7	25.6 ± 0.47	15.2 ± 0.4	9.8 ± 0.4

Values represent the average of three replicates ± standard deviation.

Also, decrease in the activity of NCIM 5802 keratinase was noticed upon treatment with EDTA (metallo-protease inhibitor), urea, β-mercaptoethanol (reducing agent), and PMSF (serine protease inhibitor). The keratinolytic protein was severely inhibited in the presence of PMSF (residual activity: 5.28%). EDTA, Urea and β-ME had inhibitory effects on NCIM 5802 keratinase, causing the loss of the enzyme activity by 47.3–71.1%. Treatment of the keratinase with surfactants (Triton X 100, Tween 80, SDS) and organic solvents also revealed their inhibitory effect (Table 1).

### Multi-scale characterization of degraded feathers and feather hydrolysate

#### Structural changes: profiling the feather degradation

**Figure 3:**
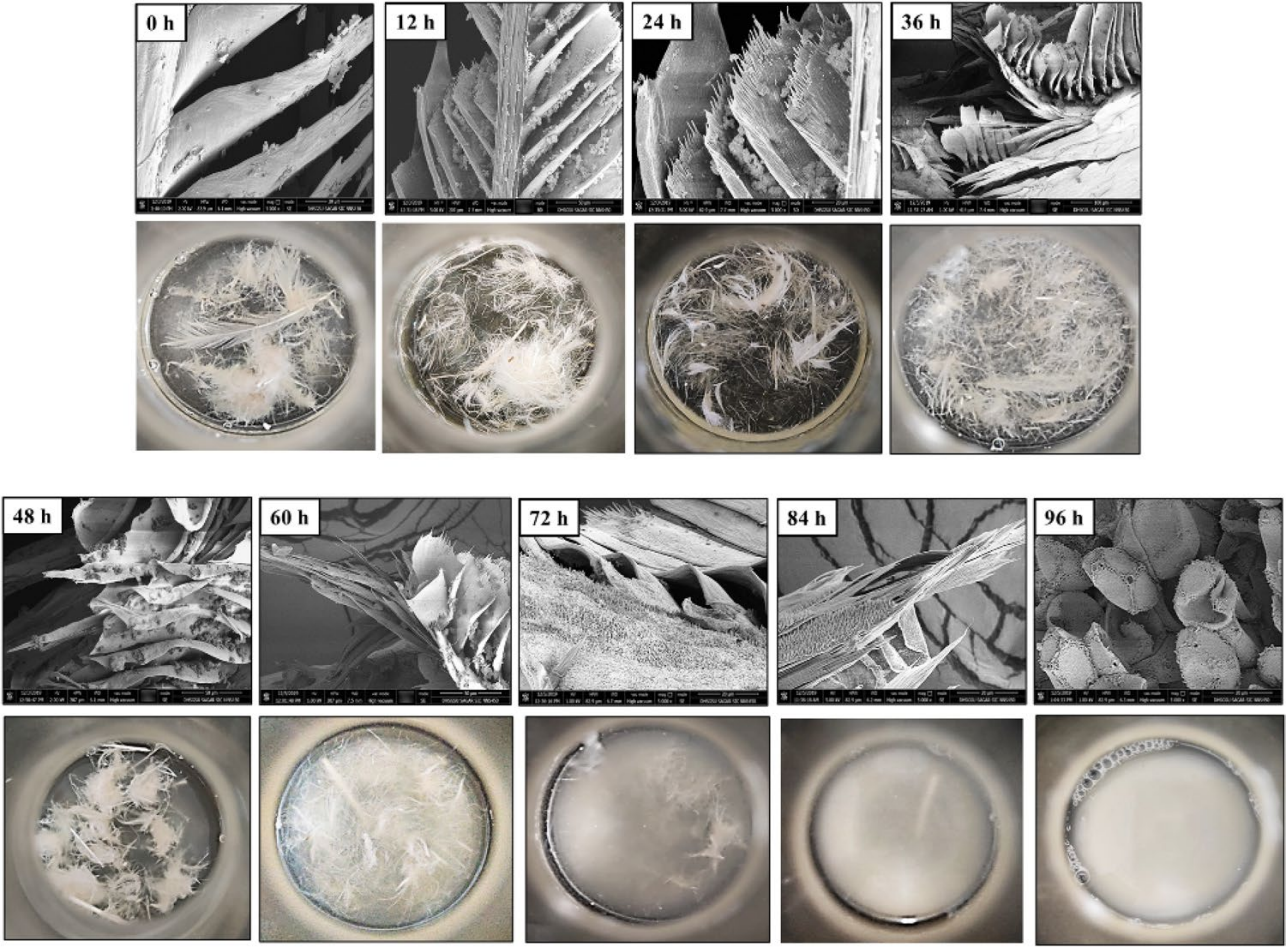
Feather degradation by *B. velezensis* NCIM 5802. Control (0 h); colonization of bacterial cells on feather surface (12–24 h); initialization of vane degradation (36 h); curling and exposure of secondary fibers (48–60 h); degradation of feather barbs, barbules, and invasion of bacterial cells in the shaft (72–84 h); complete disruption of feather rachis and shaft (96 h) by the colonized bacteria. Feather samples were harvested from the submerged cultures, gold coated and viewed at 5000 X in FEI Scanning electron microscope.

Microstructural and topological changes in the feather keratin before and after bacterial degradation were investigated by scanning electron microscopy (SEM). Distinct stages of disintegration and biodegradation of chicken feather due to keratinase action were noticed (Fig. 3). Uninoculated feathers retained the highly ordered form and crystalline structure with tiered branched assemblies of barbules, barbs, rachis and shaft whereas, the bacterial colonization on feather barbs and disintegration was clearly noticed in case of feathers inoculated with bacterial culture. Considerable fracturing, complete degradation of barbules, and invasion of bacterial cells towards the keratin fibrils was observed during 36–48 h (Fig. 3). Complete degradation of barbs and barbules was recorded after 84 h-96 h of incubation.

#### FTIR analysis of treated feathers

**Figure 4:**
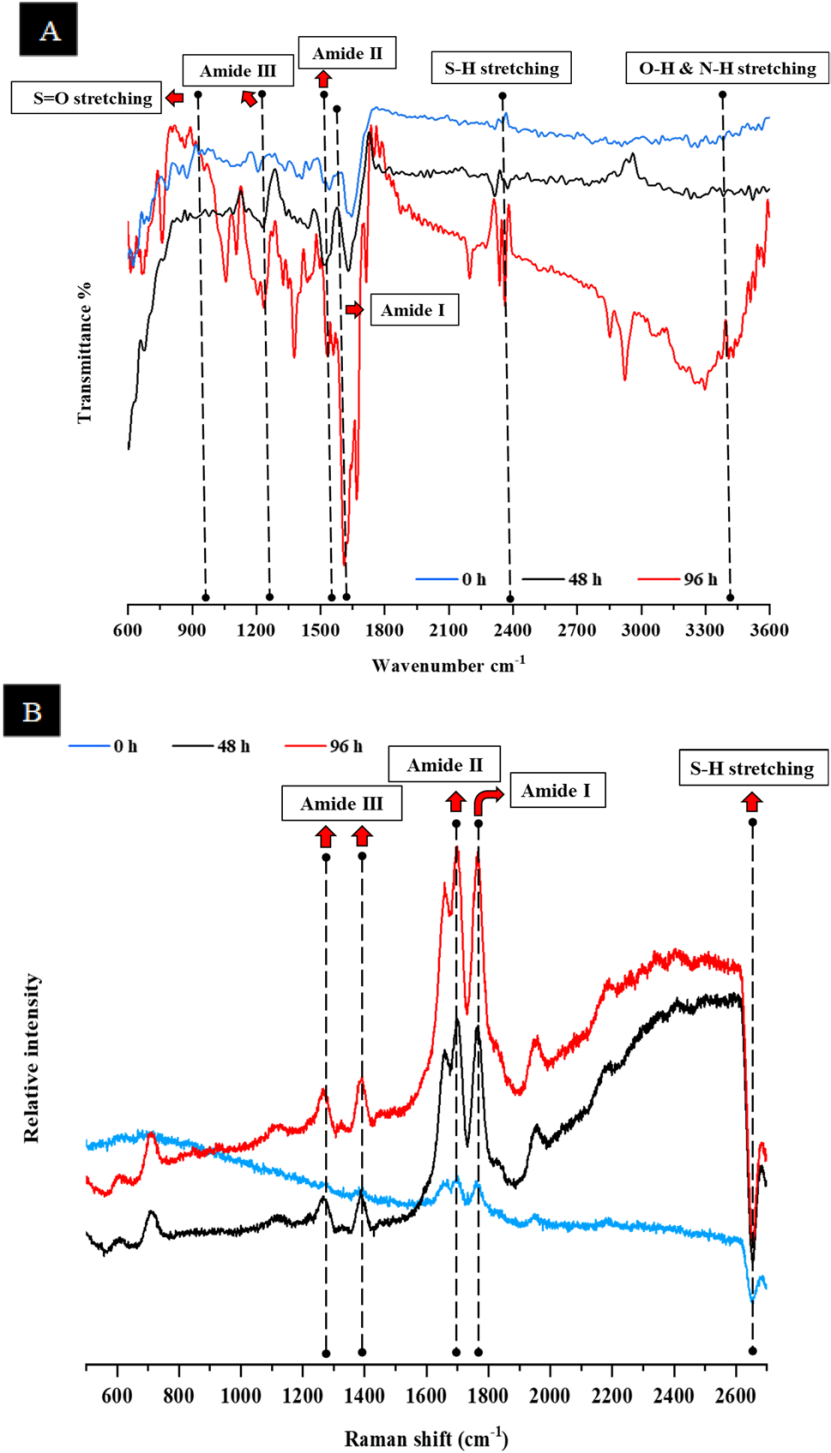
(**A**) Functional group characterization and structural changes in intact (blue) and hydrolyzed feather samples at 48 h (black) and 96 h (red) using ATR-FTIR spectra. (**B**) Chemical fingerprinting of hydrolyzed and unhydrolyzed chicken feathers using Raman spectra of intact feather (blue) and hydrolyzed feathers at 48 h (black) and 96 h (red) incubation.

The FTIR spectra of the unhydrolyzed and hydrolyzed feather keratin revealed the presence of characteristic absorption peak assigned to CONH, i.e., peptide bonds. The enzyme treated feather exhibited a broad absorption band for Amide A which is related to the secondary structure of feather (3250–3400 cm^−1^) linked to O–H and N–H stretching, Amide I (1600–1700 cm^−1^) showed an intense band for the α-helical structure corresponding to C=O stretching vibration, Amide II (1480–1580 cm^−1^) marked the presence of C−N and N−H stretching (Fig. 4A). Presence of α-helical and β-sheet conformation in feather keratin (1570–1720 cm^−1^) and the broad transmission band in the range of 1220–1300 cm^−1^ were accredited to C=O, N–H bending, and C–N stretching in-phase combination, respectively, which are identified as Amide III. Furthermore, the presence of an intense, sharp band at 600–1000 cm^−1^ indicated the integrity of the disulfide bonds. Additionally, the presence of a weak intensity peak at 1024 cm^−1^ in the hydrolyzed feather indicated the symmetric S=O stretching of the Bunte salt residues which were not observed in the unhydrolyzed feather sample.

#### Raman spectroscopic studies of the keratinase treated feathers

Chemical fingerprinting of native and treated chicken feathers was obtained using Raman spectroscopy. The graph presented in Fig. 4B, portrays these changes as a function of incubation time, where the spectral intensity of Amide I was observed at 1768 cm^−1^, Amide II at 1600–1700 cm^−1^, Amide III at 1400–1200 cm^−1^ corresponded to C–H stretching and S–S stretching vibration at 600 cm^−1^ is linked with cystine residues. The vibration of the thiol inorganic anion associated with S–H stretching vibration is related to cysteine (–S–SH or –S–S–SH) at 2600–2700 cm^−1^. The spectral peak of Amide I suggested that the degraded keratin remained largely in the β-sheet conformation whereas, the Amide III component suggested the presence of random coil and β-sheet conformation of the protein.

#### X-ray diffraction (XRD) analysis of hydrolyzed feather keratin

**Figure 5:**
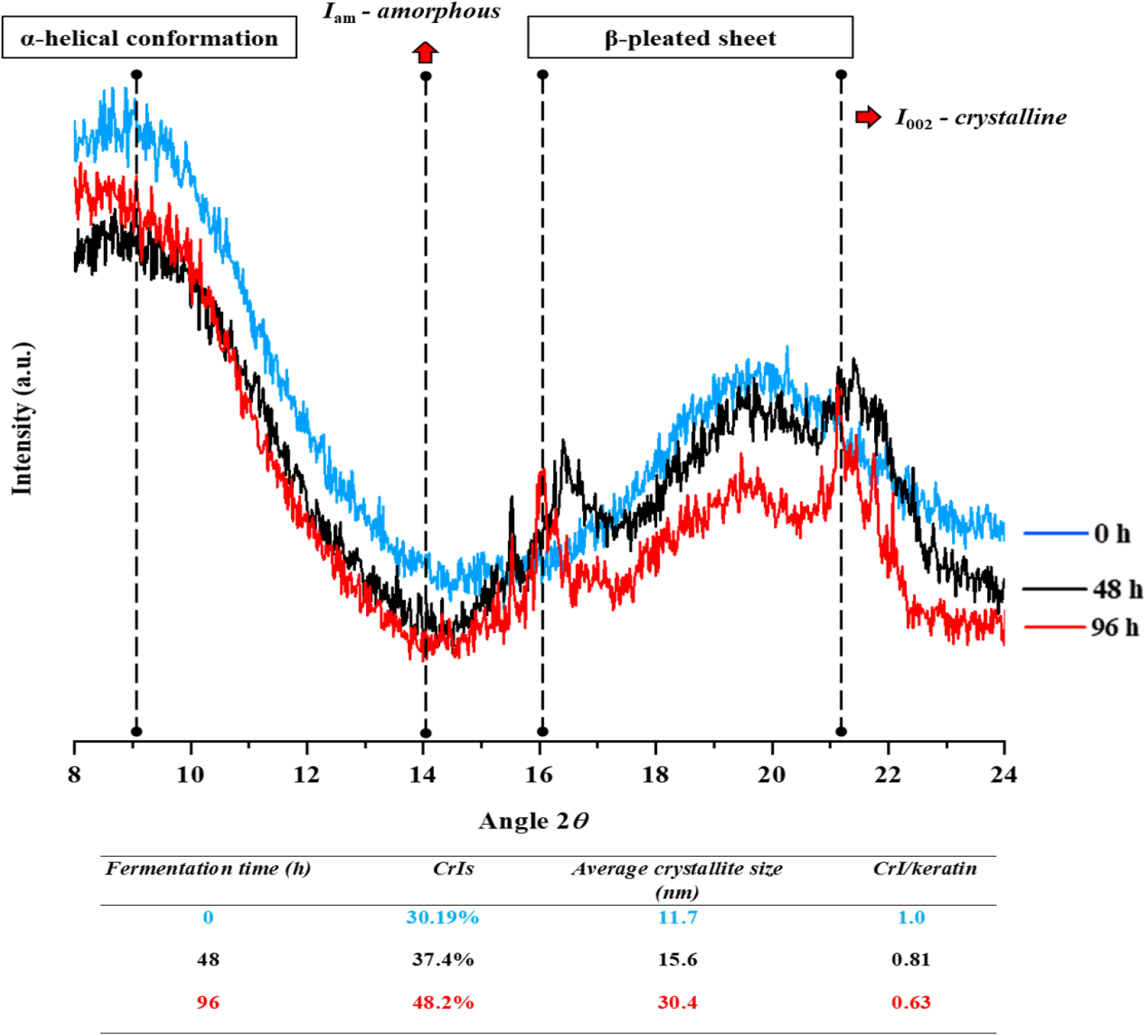
X-ray diffraction patterns of the intact (blue) and hydrolyzed feather samples at different time intervals 48 h (black) and 96 h (red) of incubation.

XRD analysis was performed to examine the crystal structures at various stages of chicken feather degradation during the course of fermentation. Figure 5 shows the XRD pattern of the native and treated chicken feathers at different time intervals. The diffraction peak at 2*θ* of about 9.0° corresponding to the α-helical conformation, and the peaks at 16.0° and 21.0°, indexed as the crystalline structure of antiparallel-pleated sheet, were observed for the raw feather (at 0 h). In comparison to raw keratin, the intensity of the broader peaks decreased remarkably in the hydrolyzed feathers, and several peaks emerged in the subsequent duration of fermentation that could be attributed to the amorphous structure of feather keratin. The CrI of treated feathers after successive incubation (37.4% and 48.24%) was higher than the CrI of untreated feathers (30.19%) (Fig. 5). Hence, the CrI of treated feathers after 96 h of incubation was 18.05% higher than the raw unhydrolyzed feather. Relative CrI (CrI/keratin) of keratin polymer after 96 h incubation was found to decrease in the treated feathers (0.63) as compared to raw keratin (1.0). Consistent with these results, the crystallite sizes (D_SE_) of the native and treated chicken feathers were calculated. The major diffraction peaks at 2*θ* =  ~ 16, 21, and 22° were employed in samples of hydrolyzed and unhydrolyzed feathers for the evaluation of crystallite sizes. Raw unhydrolyzed keratin showed the D_SE_ of about 11.7 nm and hydrolyzed feather after 96 h of incubation showed the highest D_SE_ about 30.4 nm.

#### Amino acid profile of the feather hydrolysate

Amino acid content in the feather hydrolysate i.e., the cell-free culture supernatant withdrawn at regular time intervals up to 96 h of incubation, is given in Table 2. It shows the types of amino acid profiles noticed at different time periods. Moreover, the presence of particular amino acids at different time intervals was also monitored e.g., after 12 h of incubation, arginine, serine, and threonine were observed. Along with these proline, valine, leucine, isoleucine, and asparagine were detected after 24 h of incubation. Afterwards, at the completion of 36 h incubation time histidine, phenylalanine, methionine, lysine, cysteine and tyrosine were observed, whereas after 48 h aspartate, glutamine, glutamate and tryptophan were noticed. Additionally, at 72 h alanine was released in the feather digest. After the completion of incubation period i.e., at 96 h about 18 free amino acids were detected in the feather hydrolysate.

**Table 2:** Free amino acid composition of feather hydrolysate produced by *B. velezensis* NCIM 5802 at different time intervals as analyzed by ESI–MS.

	Fermentation time (h)							
	12	24	36	48	60	72	84	96
**Amino acid (Da)**	Ser—106.1	Thr—118.9	Thr—118.9	Thr—118.9	Thr—118.9	**Ala—89.1**	Thr—118.9	Thr—118.9
	Thr—118.9	Ser—106.1	Pro—116.01	Pro—116.01	Pro—116.01	Thr—118.9	Pro—116.01	Pro—116.01
	Arg—175.4	**Pro—116**	Asn—130.9	Asn—130.9	Asn—130.9	Pro—116.01	Asn—130.9	Val—117.1
		**Val—117.1**	Leu—131.9	Leu—131.9	Leu—131.9	Asn—130.9	Leu—131.9	Asn—130.9
		**Asn—130.9**	Ile—132	Ile—132	Ile—132	Leu—131.9	Ile—132	Leu—131.9
		**Leu—131.9**	Ser—106.1	Ser—106.1	Ser—106.1	Ile -132	Ser—106.1	Ile—132
		**Ile—132**	Arg—175.4	**Asp—135**	Asp—135	Ser—106.1	Asp—135	Ser—106.1
			**Lys—146.2**	**Glu—145.4**	Glu—145.4	Asp—135	Glu—145.4	Asp—135
			**Cys—122.5**	**Gln—149**	Gln—149	Glu—145.4	Gln—149	Glu—145.4
			**His—156**	**Trp—206.4**	Lys—146.2	Gln—149	Lys—146.2	Gln—149
			**Phe—162.5**	Lys—146.2	Arg—175.4	Lys—146.2	Arg—175.4	Lys—146.2
			**Tyr—181.8**	Arg—175.4	His—156	Arg—175.4	His—156	Arg—175.4
			**Met—149.2**	His—156	Phe—162.5	His—156	Phe—162.5	His—156
				Phe—162.5	Tyr—181.8	Phe—162.5	Tyr—181.8	Phe—162.5
				Tyr—181.8	Met—149.2	Tyr—181.8	Met -149.2	Tyr—181.8
				Met—149.2	Cys—122.5	Met—149.2	Cys—122.5	Met—149.2
				Cys—122.5	Trp—206.4	Cys—122.5	Trp—206.4	Cys—122.5
						Trp—206.4		Trp—206.4

Significant values are given in bold.

#### NMR spectroscopic studies: generation of amino acids from polymeric substrate

**Figure 6:**
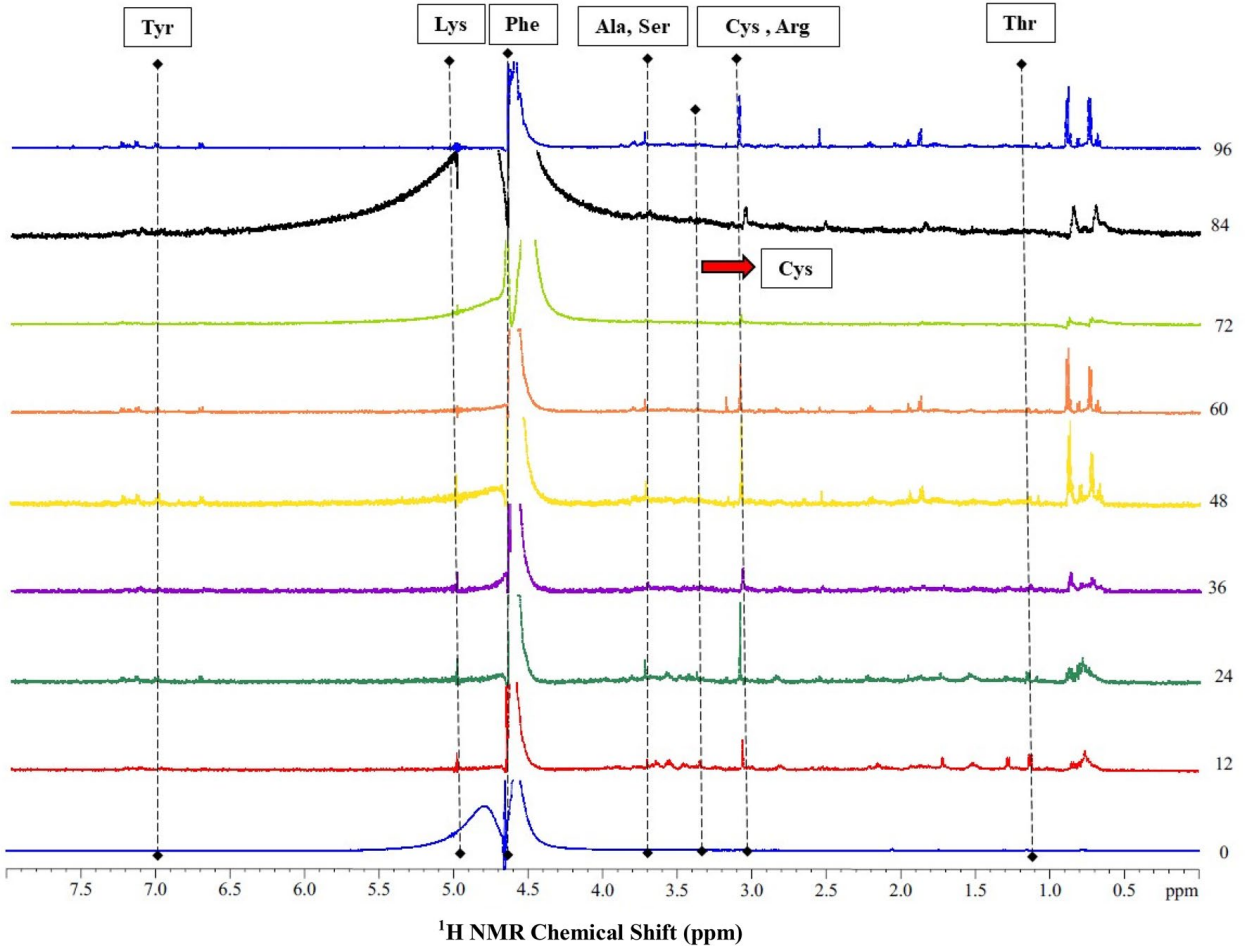
^1^H NMR spectra showing decomposition of native chicken feathers during the cultivation time of 96 h by *B. velezensis* NCIM 5802 and the release of amino acids.

The ^1^H NMR spectra of the feather hydrolysates showing presence of various amino acids is presented in Fig. 6. The absorption spectral peak at 1.3 ppm indicated the presence of threonine (Thr), while those at 3.2 ppm and 3.5 ppm were attributed to cystine (CH_2_–S–S), arginine (Arg) and cysteine (CH_2_–S–H, Cys). Peaks at 1.5 ppm, 4.0 ppm, 4.8 ppm and 7.0 ppm were assigned to alanine (Ala), serine (Ser), lysine (Lys) and tyrosine (Tyr), respectively. Also, the broad peak at 4.6 ppm was assigned to phenylalanine (Phe). Thus, the ^1^H NMR of liquid hydrolysate confirmed the release of amino acids indicating the depolymerization of feather keratin by *B. velezensis* NCIM 5802.

#### Zymographic profile of the feather hydrolysate

**Figure 7:**
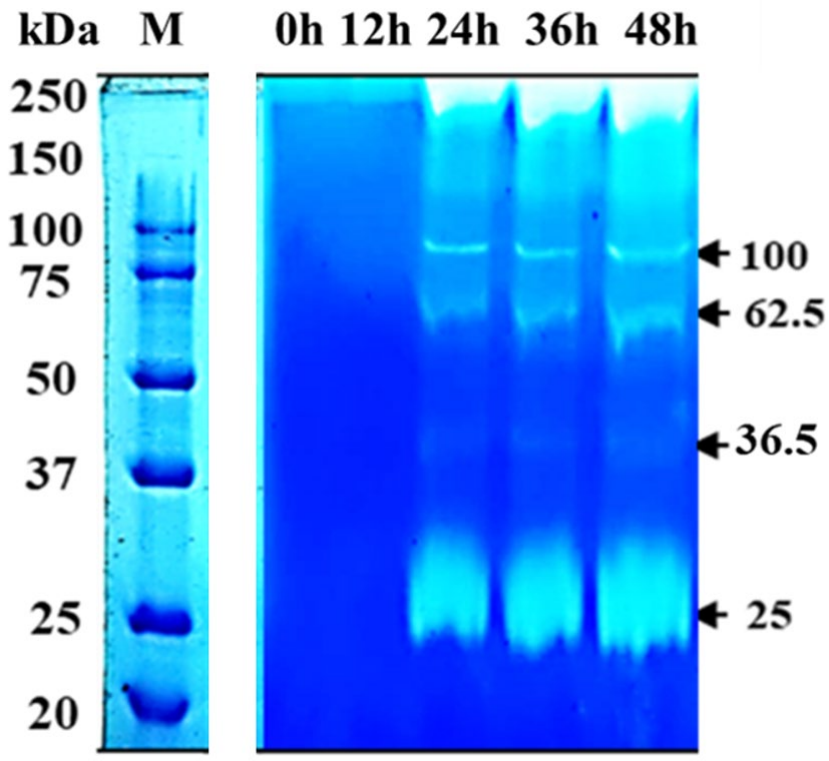
Keratinase zymogram showing NCIM 5802 keratinase during the course of fermentation at different time intervals (0 h-48 h), M: standard protein marker. The semi-native PAGE contained 1% feather keratin. The gel was stained with Coomassie Blue after 24 h of incubation at 40 °C.

The bacterium was observed to produce the active keratinases after 24 h of fermentation time (Fig. 7) as no keratinase activity was detected after the 12 h of incubation whereas, the presence of four distinct keratinases (Mr ~ 100, 62.5, 36.5, and 25 kDa) was noticed indicating secretion of multiple keratin hydrolyzing enzymes by *B. velezensis*.

## Discussion

Recent advancements in biomass valorization research suggest that apart from lignocellulose and chitin, keratin will also play a major role in bioeconomy^20,37^. Feather keratin, in particular, consists mainly of β-pleated sheets and due to its recalcitrant nature, robust enzymes of fungal or bacterial origin are needed for its degradation^37^. Fungal keratin degradation process appears to be relatively slow while some bacterial species including *B. licheniformis* RG1^38^, *Fervidobacterium pennavorans*^39^, *Fervidobacterium islandicum* AW-1^40^, *Meiothermus taiwanensis* WR-2230^41^ and *Bacillus cereus* IIPK35^29^ are known to produce high titers of keratinases in about 30–120 h. In the present study, *B. velezensis* NCIM 5802 was found to produce keratinase and degrade feather keratin completely in 84–96 h of fermentation. *B. velezensis* NCIM 5802 efficiently degraded chicken feathers and the keratinase activity reached to highest level in 96 h of fermentation.

Hossain et al.^42^ reported that in the absence of any redox reaction, about 10–20% of feathers are degraded solely by bacterial keratinases. Keratinases derived from *B. licheniformis* ER-15, *Bacillus paralicheniformis* MKU3 and *B. cereus* IIPK35 degraded around 90% of the feather keratin^29,43,44^. In the present study, newly isolated *B. velezensis* NCIM 5802 degraded feathers completely (~ 99.1%) into amino acids and soluble proteins. In order to achieve dual targets of effective feather degradation and maximum keratinase production, the parametric optimization of both the processes was achieved through statistical optimization using CCD. The optimal conditions (40 °C, pH 8.0, substrate load 0.625% and inoculum size 3.5%) were validated resulting in the enhancement of both, feather degradation and keratinase production by 2.5 and 4.92-folds, respectively. These results are in agreement with those obtained by Kshetri et al.^10,^ obtaining 84% of feather degradation along with the increased production of soluble peptides after statistical optimization. After optimization of process parameters Jana et al.^29^ achieved five fold enhanced titers of keratinase.

A follow-up of the time course of bacterial growth on feathers showed a pH shift from neutral to alkaline (7.0–9.5). Peng et al.^45^ have suggested that alkaline environment or a reducing agent is required for the breaking of the disulfide bonds, and as keratin degradation is a complex process, it requires a combination of enzymes mainly, disulfide reductase, cysteine dioxygenase (EC 1.13.11.20), glutathione reductase (EC 1.8.1.7), thioredoxin reductase (EC 1.8.1.9) for efficient degradation of feathers^26,45,46^. Simultaneous to the enzymatic degradation of the feathers, corresponding increase in the soluble protein (8398.5 μg/mL), amino acid (1151.8 μg/mL), and oligopeptide (930.77 μg/mL) content was noticed after 84–96 h of fermentation.

The keratinase activity profile signifies a broad pH working range (7.0–11.0) with an optimum at 10.0. The keratinase was active in the temperature range of 30 to 90 °C with optimal activity at 60 °C. These cardinal values are in agreement with earlier reports showing that the optimal pH value of alkaline keratinase is close to 10.0^47–50^ (Table 3). Similarly, several researchers have reported the optimal functioning of the *Bacillus* keratinases (Table 3) in the range of 60–65°C^25,51^.

**Table 3:** Comparative account of characteristics of bacterial keratinases.

Bacteria	Optimum		Catalytic type	Activators	Inhibitors	References
	pH	Temp. (°C)				
*Ochrobactrum intermedium*	9.0	40	Metallo-serine	K^+^, Na^+^, Ca^2+^ and Mg^2+^	PMSF, EDTA, Zn^2+^, Fe^2+^, Cu^2+^, and Mn^2+^	Sharma and Kango^23^
*Bacillus licheniformis* ALW1	8.0	65	–	–	–	Abdel-Fattah et al.^25^
*Bacillus cereus*	7.0–9.0	30–45	Metallo	–	–	Verma et al.^28^
*Bacillus* sp.	7–8	30–37	Serine/metallo	–	–	Verma et al.^28^
*Laceyella sacchari* YNDH	10.4	70	Mixed Serine/metallo	PMSF, EDTA, DTT	Cu^2+^, Mn^2+^, Zn^2+^, and Co^2+^	Goda et al.^47^
*Bacillus* sp.	11.0	60	Serine	–	PMSF, H_2_O_2_, Ca^2+^	Genckal and Tari^48^
*Bacillus clausii*	11.0	60	Serine	SDS, H_2_O_2_	PMSF, Leupeptin	Joo et al.^52^
*Bacillus subtilis* DP1	10.0	37	Serine	Ca^2+^ and Mg^2+^	PMSF, EDTA Mn^2+^ and Zn^2+^	Sanghvi et al.^58^
*Paenibacillus woosongensis* TKB2	9.0	50	Serine	Mo^+^, Fe^2+^ and Mn^2+^	PMSF, SDS, EDTA and β-ME	Paul et al. ^60^
***B. velezensis* NCIM 5802**	**10.0**	**60**	**Metallo-serine**	**Ca**^**2+**^** and Mg**^**2+**^	**EDTA, PMSF, Ag**^**2+**^** Hg**^**2+**^** and NaN**_**3**_	**This study**

Significant values are given in bold.

The keratinase retained more than 60% activity at pH 10.0 after 1 h, indicating its alkali stable nature. In this context, *Bacillus subtilis* keratinase has been shown to be stable in the pH range of 10–12^48,52^. Being able to retain 80% activity at 60 °C, the keratinase was considered thermostable. Elevated temperatures are known to increase the rate of keratinolysis^47^. Our findings are in agreement with those reported for *B. licheniformis* ALW1 thermo and alkali stable keratinase^25^.

The NCIM 5802 keratinase activity was strongly inhibited by PMSF and EDTA indicating it being a metalo-serine protease. EDTA removes the metal ion through chelation, suggesting the requirement of metal ion(s) for the proficient enzyme catalysis^28,53,54^. Presence of Ca^2+^ and Mg^2+^ boosted the enzyme activity by 53.8% and 111.8%, respectively. This upsurge in the enzymatic activity in the presence of divalent metal ion(s) suggested that the active conformity of the enzyme is maintained by these cations, which in turn, contributes to the increased keratinolytic activity. As this enzyme belonged to the class of serine-proteases, Ca^2+^ may play an important role in the stabilization of the enzyme structure and function^23,55,56^. Our findings suggest that these metal ion(s) possess a functional role in the structural stabilization of the enzyme, meaning that studied metal ions ensure the proper binding of the enzyme–substrate complex at the active site. All other metal ions (K^+^, Na^+^, Zn^2+^, Fe^2+^, Cu^2+^, Mn^2+^) moderately inhibited the keratinase activity^57^. Likewise, Ag^+^, Hg^2+^ and NaN_3_ drastically decreased the keratinase activity, which implies that a cysteine residue in the free form is present at the active site of the enzyme^57^. Our findings are in agreement with several authors, who reported the inhibitory effect of such metal ions on the keratinase^24,57,58^.

On the other hand, reducing agents, non-ionic surfactants, and solvents were found to inhibit keratinase, which is in accordance with the findings reported earlier^58–60^. Literature suggests that non-ionic surfactants or solvents disintegrate the keratinase structure by partly solubilizing it^61^.

The sequential changes occurring during feather keratin hydrolysis were investigated through multiscale analyses. Scanning electron microscopy revealed bacterial adherence and colonization of the feathers and damage in the barbs, that marked the first step of degradation followed by the extensive fracturing and disintegration of barbs and barbules. Subsequently, the bacterial invasion, decomposition, and weakening of the most recalcitrant part of the feather i.e., rachis was observed. Afterwards, the final stage of keratinolysis resulted in the complete disintegration of the amorphous porous keratinous material. Among several studies employing bacteria for feather keratin degradation, the time course for the complete degradation of keratin varied from 1 to 6 days^29,40,56,62^. Shorter span of time is favorable for industrial applications and few reports, including the present study, demonstrate the efficient keratin hydrolysis within 24–96 h^23,26^. We have reported a process of keratin degradation with an efficiency exceeding 95% in 84 h.

To understand the dynamics of keratin biodegradation and structural changes in the keratin at molecular level, FTIR and Raman spectroscopic analyses were conducted where, the changes in the intensity and position of the bands are attributed to the conformational changes in the keratin structure. The spectral peak of Amide I and Amide II at 1600–1700 cm^−1^ and 1480–1580 cm^−1^ respectively, corresponds to the α-helical and β-sheet structure in feather keratin. In the hydrolyzed feather keratin, the peak intensity of β-sheet structure present in the initial phases was reduced with the time of incubation along with the shift in –NH stretching region signifying the potential degradation of the keratin fibers^63^. The degradation of sulfide-containing cysteine was more evident by the weak S=O bond at 1024 cm^−1^. The increase in the intensity of stretching vibrations at 560–600 cm^−1^ suggested that a significant amount of the -S–S- bonds in the protein have been cleaved indicating the weakening of the keratin structure. The microbial degradation of the keratin was more evident as the peak of the S–H (thiol) bond at 2400 cm^−1^ was intensified with the increase in the fermentation time. Changes in the secondary structure and reduction in disulfide bonds with thiol (S–H) formation with respect to cultivation time indicated the proteolytic and sulfitolytic prospects of the isolate, which is an indicator of efficient keratin hydrolysis^34,36^. The results of FTIR and Raman analysis complemented each other where, distinct changes in the vibrations of the hydrolyzed and unhydrolyzed feather keratin were noticed^63,64^.

The relative crystallinity index (CrI) is considered as one of the major attributes that affects the hydrolysis kinetics of keratin. Relative CrI of keratin (0.63) was found to decrease in the hydrolyzed feather keratin as compared to raw keratin (1.0) after 96 h incubation. High crystallinity index represents the tight packaging of β-pleated sheets in feather keratin whereas, the reduction in relative crystallinity index of hydrolyzed feather keratin along with the increase in the crystallite size with the incubation time indicates the breakdown of disulfide bonds thus, successful keratin hydrolysis^35,65^. Crystallinity of the macromolecule plays an important role in maintaining the integrity, stability, providing strength to the keratin and resistance to proteolysis^66,67^.

Furthermore, the present report of biodegradation and enzymatic conversion of feathers into value-added by-products is purely an enzymatic process that is not only simple and streamlined but also inexpensive, in which no additional chemicals are supplemented to aid the feather degradation. The amino acids present in feather hydrolysate were studied through ESI–MS and, about 18 free amino acids were detected (Table 2) in consort with the rare amino acids, including serine and proline, and sulfur-containing amino acids including methionine^47,68^. Likewise, the presence of amino acids in the hydrolysates was also investigated through ^1^H NMR spectroscopy, where a number of amino acids was detected with respect to the cultivation time. The result is in accordance with the findings reported by Barone and Schmidt^36^.

Additionally, to investigate the nature of the enzyme responsible for the keratin hydrolysis the keratinase activity was detected on the gel and four distinct bands (Mr ~ 100, 62.5, 36.5 and 25 kDa) of keratin hydrolysis were observed. Multiplicity of keratinases (six bands ranging from 17 to 122 kDa) has been reported from *Bacillus* sp.^69^ and our findings are in agreement with the earlier reports^47,70^. Bacterial keratinases are known to occur in wide range of molecular mass, e.g., 18 kDa in *Streptomyces albidoflavus,* while 240 kDa in *Kocuria rosea*. Keratin being a complex, recalcitrant molecule may require endoprotease, exoprotease, and oligopeptidase activities to work synergistically^71^. The present work suggested that the feather keratin can be transformed into usable products including, feather meal, amino acids, or protein concentrates by employing the keratinases derived from newly isolated *B*. *velezensis*.

## Conclusion

The keratinolytic potential of the newly isolated *B. velezensis* NCIM 5802 producing thermo-alkali-stable keratinase consortia was evaluated. It effectively degraded the feather keratin with an efficiency of 99.1% along with the production of soluble protein (8398.5 μg/mL), amino acids (1151.8 μg/mL) and oligopeptides (930.77 μg/mL) in 96 h. The extracellular keratinase preparation was found to be a serine-metallo protease showing optimal activity at 60 °C and pH 10.0. The biodegradation of feather keratin was mediated by multiple keratinases as revealed by the zymography. Multi-scale spectroscopic analysis of treated feathers using FTIR, Raman, XRD and NMR revealed the sequential degradation of β-sheets and α-helices along with disulfide bonds. The results presented in this paper provide new insights into the feather keratin degradation and potential of *B. velezensis* NCIM 5802 in poultry waste valorization for animal feed production and effective management of keratin waste.

## Supplementary Information

Supplementary Information.

## Data Availability

The 16S rRNA sequences of *Bacillus* sp. NCIM 5802 were submitted to the NCBI accession number ON203026 (https://submit.ncbi.nlm.nih.gov/subs/?search=SUB11319989) and the bacterial culture is submitted to NCIM accession number NCIM-5802 (https://www.ncl-india.org/files/ncim/CatalogueDetails.aspx?NCIMNo=5802). The authors declare that all data supporting the findings of this study are available within the article.

## Abbreviations

FMMSA: Feather meal minimal salt agar
RSM: Response surface methodology
CCD: Central composite design
ANOVA: Analysis of variance
SD: Standard deviation
BSA: Bovine serum albumin
EDTA: Ethylenediaminetetraacetic acid
PMSF: Phenylmethylsulfonyl fluoride
β-ME: β-Mercaptoethanol
SDS: Sodium dodecyl sulfate
ESI–MS: Electrospray ionization–mass spectrometry
CID: Collision induced dissociation
ELS: Evaporative dissociation light scattering
SEM: Scanning electron microscope
FTIR: Fourier transform infrared spectroscopy
NMR: Nuclear magnetic resonance
TMS: Tetramethylsilane
XRD: X-ray powder diffraction
