# Supplementary material for: Parametrically optimized feather degradation by Bacillus velezensis NCIM 5802 and delineation of keratin hydrolysis by multi-scale analysis for poultry waste management
Source: Sci Rep. 2022 Oct 12;12:17118. doi: 10.1038/s41598-022-21351-9 (PMC9556542; doi:10.1038/s41598-022-21351-9)

| *Run* | *Factor* | | | | *Response* | | | |
| --- | --- | --- | --- | --- | --- | --- | --- | --- |
|  | **Temperature (℃)** | **pH** | **Substrate concentration (%)** | **Inoculum size (%)** | **Keratinase activity**  **(U/mL)** | | **Feather Degradation (%)** | |
|  | **A** | **B** | **C** | **D** | **Values** | | | |
|  |  |  |  |  | **Predicted** | **Actual** | **Predicted** | **Actual** |
| 1 | 50 | 9 | 1.0 | 6.0 | 27.2 | 22.2 ± 0.1 | 64.2 | 88.0 ± 0.05 |
| 2 | 50 | 7 | 1.0 | 6.0 | 24.6 | 22.9 ± 0.5 | 61.8 | 39.0 ± 0.06 |
| 3 | 30 | 7 | 1.0 | 6.0 | 30.1 | 26.2 ± 0.3 | 44.2 | 55.0 ± 0.3 |
| 4 | 40 | 8 | 0.6 | 1.5 | 86.1 | 88.3 ± 0.5 | 66.8 | 91.8 ± 0.8 |
| 5 | 40 | 6 | 0.6 | 3.5 | 30.7 | 49.8 ± 0.2 | 39.9 | 69.7 ± 1.3 |
| 6 | 50 | 9 | 0.2 | 6.0 | 29.5 | 32.3 ± 0.1 | 21.0 | 20.8 ± 5.2 |
| 7 | 30 | 7 | 0.2 | 6.0 | 45.1 | 43.3 ± 0.6 | 40.3 | 39.2 ± 0.09 |
| 8 | 60 | 8 | 0.6 | 3.5 | 5.6 | 16.5 ± 2.4 | 1.51 | 8.0 ± 2.0 |
| 9 | 40 | 10 | 0.6 | 3.5 | 22.0 | 23.2 ± 1.3 | 15.2 | 28.7 ± 0.05 |
| 10 | 30 | 9 | 0.2 | 6.0 | 43.7 | 42.6 ± 0.2 | 31.5 | 48 ± 0.02 |
| 11 | 40 | 8 | 0.1 | 3.5 | 22.2 | 23.1 ± 0.3 | 18.9 | 19.9 ± 0.04 |
| 12 | 40 | 8 | 0.6 | 3.5 | 102.9 | 104.5 ± 0.8 | 90.7 | 93.4 ± 1.8 |
| 13 | 40 | 8 | 0.6 | 3.5 | 102.9 | 100.9 ± 0.05 | 90.7 | 100 ± 0.07 |
| 14 | 50 | 7 | 0.2 | 6.0 | 20.9 | 18.6 ± 0.1 | 11.7 | 4.0 ± 2.3 |
| 15 | 50 | 9 | 0.2 | 1.0 | 18.9 | 17.0 ± 3.2 | 21.9 | 4.0 ± 0.8 |
| 16 | 30 | 7 | 1.0 | 1.0 | 12.3 | 7.2 ± 2.2 | 22.2 | 1.7 ± 1.7 |
| 17 | 30 | 9 | 1.0 | 1.0 | 19.5 | 20.2 ± 0.8 | 23.4 | 54.3 ± 2.2 |
| 18 | 50 | 7 | 0.2 | 1.0 | 22.1 | 23.1 ± 0.03 | 44.4 | 48.8 ± 0.27 |
| 19 | 40 | 8 | 1.4 | 3.5 | 38.7 | 59.9 ± 1.3 | 41.5 | 57.8 ± 1.2 |
| 20 | 40 | 8 | 0.6 | 3.5 | 102.9 | 128.3 ± 2.3 | 90.7 | 97.2 ± 2.7 |
| 21 | 40 | 8 | 0.6 | 8.5 | 42.9 | 64.4 ± 0.02 | 79.6 | 96.0 ± 0.3 |
| 22 | 30 | 7 | 0.2 | 1.0 | 43.9 | 45.8 ± 0.5 | 35.5 | 37.2 ± 0.08 |
| 23 | 40 | 8 | 0.6 | 3.5 | 102.9 | 110.5 ± 4.2 | 90.7 | 94.3 ± 1.7 |
| 24 | 20 | 8 | 0.6 | 3.5 | 21.9 | 32.2 ± 0.03 | 13.5 | 20.3 ± 0.5 |
| 25 | 30 | 9 | 1.0 | 6.0 | 12.3 | 11.1 ± 1.6 | 4.51 | 0.96 ± 5.2 |
| 26 | 50 | 7 | 1.0 | 1.0 | 20.8 | 13.8 ± 0.07 | 19.1 | 5.6 ± 0.12 |
| 27 | 50 | 9 | 1.0 | 1.0 | 18.0 | 15.6 ± 0.16 | 21.7 | 8.6 ± 0.7 |
| 28 | 40 | 8 | 0.6 | 3.5 | 102.9 | 101.7 ± 1.7 | 90.7 | 94.6 ± 0.2 |
| 29 | 30 | 9 | 0.2 | 1.0 | 36.0 | 35.6 ± 0.15 | 44.4 | 48 ± 1.3 |
| 30 | 40 | 8 | 0.6 | 3.5 | 102.9 | 112.5 ± 0.06 | 90.7 | 95.2 ± 0.04 |

**Supplementary Table S1**: Experimental Central composite design (CCD) of RSM and the investigational responses for different variables with keratinase production (U/mL) and feather degradation (%). Data points indicate the average of triplicate values ± standard deviation.

**Supplementary Figure S1:** Molecular phyloanalysis based on 16S rDNA sequence, showing the relation between isolate and other species, the tree was constructed using MEGA X programme by neighbor joining tree method.

**Supplementary Figure S2:** Predicted signal peptide of *B. velezensis* revealed by SignalP 6.0 prediction server.


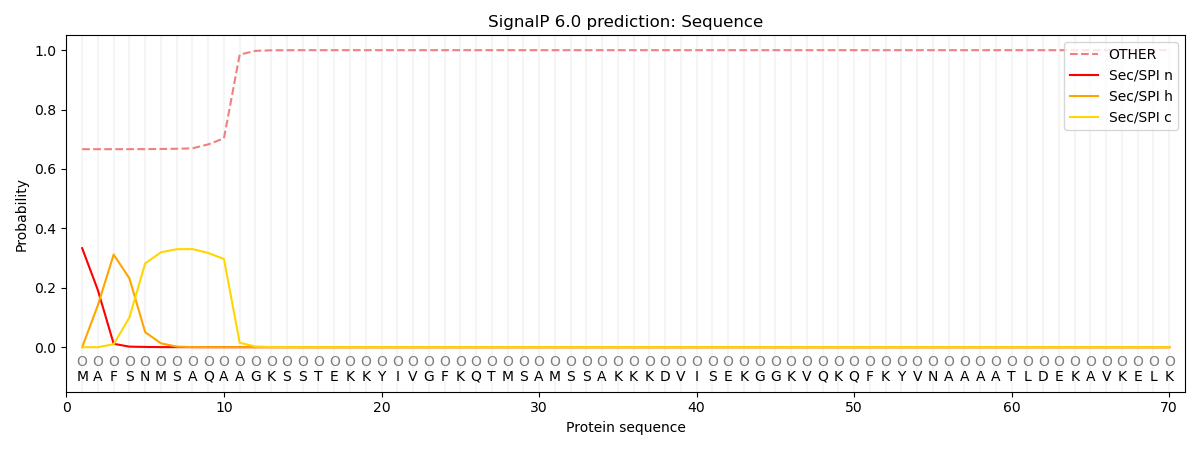

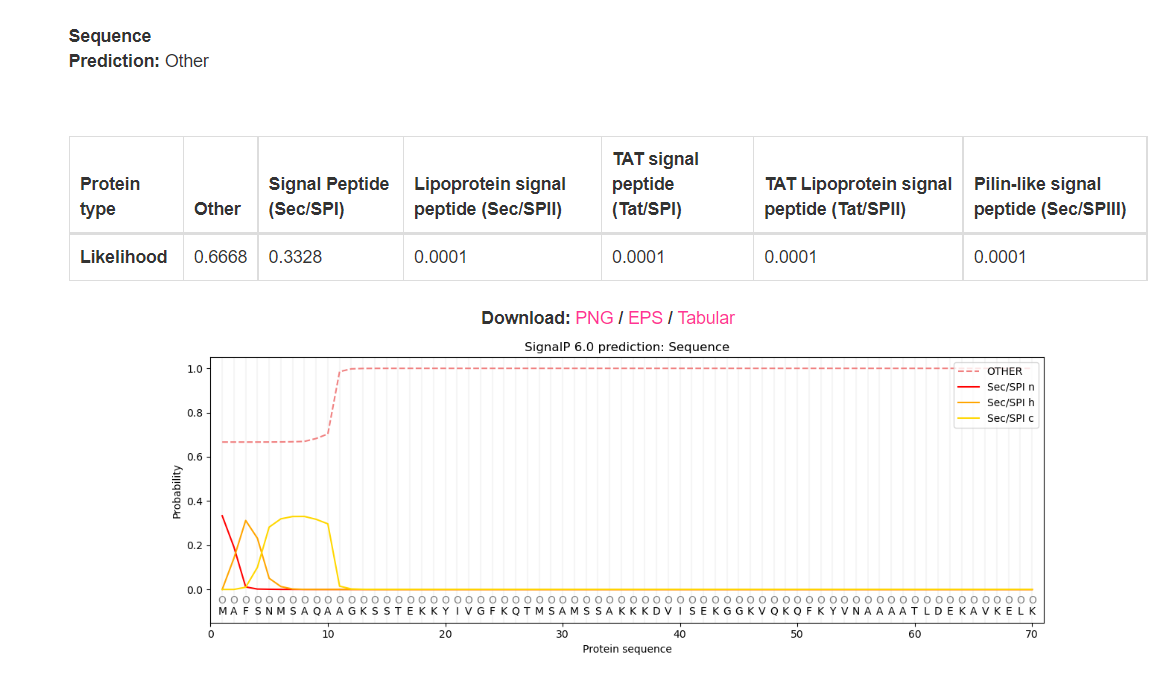


**Supplementary Figure 3:** Denaturating SDS PAGE (left) and keratinase zymogram (right) showing NCIM 5802 keratinase during the course of fermentation at different time intervals, M: standard protein marker. Zymogram contained 1% feather keratin and the gel was stained with Coomassie Blue after 24 h of incubation at 40 °C.


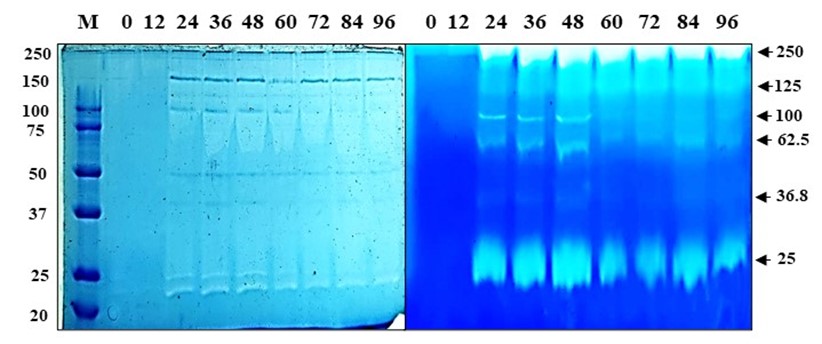

Supplement: Supplementary file 1 — Supplementary Information. [file 41598_2022_21351_MOESM1_ESM.docx]
